# Exosomes derived from pancreatic cancer cells induce insulin resistance in C2C12 myotube cells through the PI3K/Akt/FoxO1 pathway

**DOI:** 10.1038/s41598-017-05541-4

**Published:** 2017-07-14

**Authors:** Lantian Wang, Bo Zhang, Wen Zheng, Muxing Kang, Qing Chen, Wenjie Qin, Chao Li, Yuefeng Zhang, Yingkuan Shao, Yulian Wu

**Affiliations:** 1 Department of Surgery, The Second Affiliated Hospital, Zhejiang University School of Medicine, Hangzhou, P.R. China; 2 Key Laboratory of Cancer Prevention and Intervention, China National Ministry of Education, Cancer Institute, Second Affiliated Hospital, Zhejiang University School of Medicine, Hangzhou, Zhejiang P.R. China

## Abstract

Prospective epidemiological studies have consistently suggested that pancreatic cancer-associated new-onset diabetes mellitus (PC-DM) represents a potential platform for early diagnose of pancreatic cancer (PC). Despite the studies performed, the mechanism behind this phenomenon remains ambiguous. In this study, we explored the effects of two types of exosomes released by murine pancreatic cancer and ductal epithelial cells on murine skeletal muscle cells. The results show that PC-derived exosomes can readily enter C2C12 myotubes, triggering lipidosis and glucose intake inhibition. We also demonstrate that PC-derived exosomes can inhibit insulin and PI3K/Akt signalling, in which insulin-induced FoxO1 nuclear exclusion is preserved and Glut4 trafficking is impaired. Microarray and Kyoto Encyclopedia of Genes and Genomes (KEGG) analyses show that exosomal microRNAs (miRNAs) probably play key roles in this process, an assumption that is corroborated by *in vitro* studies. These results confirm that the insulin resistance (IR) of skeletal muscle cells is governed by PC-derived exosomes through the insulin and PI3K/Akt/FoxO1 signalling pathways, where exosomal miRNAs potentially contribute to this phenomenon. These novel findings pave the way towards a comprehensive understanding of the cancer theories: “metabolic reprogramming” and “metabolic crosstalk”.

## Introduction

Pancreatic cancer (PC) is one of the most invasive carcinomas worldwide^1^. The only curative therapeutic treatments rely on surgical resection, yet the efficiency of this approach is limited by the lack of inceptive diagnosis as well as highly aggressive behaviours^2^. This leads to a 5-year survival rate for PC patients that is under 7%^3^. Biochemical examinations, such as with tumour markers, and imaging examinations, which are commonly employed in the clinic, are not accurate for the diagnosis of pancreatic cancer (PC) at the early stage^4^. Therefore, the main goals of pancreatic cancer research lie in looking for markers of the early stage, understanding the biological behaviours of the tumour and developing novel therapeutic methods and targets.

Multiple clinical studies have shown that new-onset diabetes may be one of the early indicators of pancreatic cancer^5–8^. In a previous study, our group screened the diagnostic markers of pancreatic cancer-associated new-onset diabetes mellitus (PC-DM) and uncovered the underlying mechanism (Am J Gastroenterol. 2010;105(7):1661–1669; Cancer Lett. 2016;373(2):241–250). Together with recent research, we determined that pancreatic cancer not only exerts effects on pancreatic β cells, with consequent decreases in insulin secretion, but also induces a glucose uptake/utilization disorder and insulin resistance (IR) of peripheral tissues, which precedes diabetes and affects PC patients without diabetes. Inspired by PC-DM, we hope to further reveal the mechanism of the glucose uptake/utilization disorder and insulin resistance of peripheral tissues induced by PC in this study.

Insulin resistance of skeletal muscle is the main pathological component of PC-DM and type II diabetes mellitus (T2DM)^9^, under which the core factors involve the metabolic reprogramming of PC^10^. In addition to the tumour itself, one feature of metabolic reprogramming is the metabolic crosstalk between PC and peripheral tissues, based on IR. This cancer hallmark triggers a systemic metabolic disorder that has associated glucose intolerance as the initial apparent phenomenon^11^, and the subsequent IR is closely related to tumour proliferation, metastasis and cancer cachexia^12, 13^.

How does PC exert its influence on peripheral tissues from afar? The emergence of exosomes has provided a new possibility for our research in this field. Exosomes are extracellular vesicles (EVs) with diameters of 30–150 nm and are enriched in endosome-derived components^14^. When internalized by recipient cells, exosomes deliver internal bioactive components and exert regulatory effects. MicroRNAs (miRNAs) are a class of endogenous non-coding RNAs (ncRNAs). Mature miRNAs identify target mRNAs and regulate post-transcriptional gene expression^15^. Exosomes protect miRNAs from degradation induced by RNA enzymes in body fluid and transport miRNAs to recipient cells, where they participate in gene expression and signal transduction and play key roles in the processes of various diseases. There is growing evidence that the maturation process of miRNAs is linked to the formation and maturation of exosomes^16^, and exosomal miRNAs play important roles in metabolic diseases^17^, where they can be regarded as biomarkers and targets for correcting metabolism disturbances^18^.

Hence, this study aims to explore whether PC-derived exosomes induce IR of skeletal muscle cells and focuses on the biological functions of exosomal miRNAs in this process to elucidate the underlying mechanism. Our efforts will contribute to the discovery of potential targets for correcting metabolic disorders and improve diagnosis and treatment in the context of PC.

## Results

### Morphological characterization and identification of exosomes

Exosomes are extracellular vesicles (EVs) ranging in diameter from 30 to 150 nm^14^, and their morphology is typified by the hypocrateriform. An analysis of isolated microparticles that was performed using the qNano-TRPS system (Izon Science Ltd., China) and Philips Tecnai™ 10 transmission electron microscope (Philips, Netherlands) validated, by morphological characterization, the presence of exosomes with diameters of ~150 nm (Supplementary Fig. S1A–E). We also identified, by western blot analysis, the already published exosomal markers^19^ Alix and CD63. Calnexin, a ubiquitously expressed endoplasmic reticulum protein, was employed as a negative control (Supplementary Fig. S1F).

### The differentiation process of C2C12 myoblasts

The morphological nature of C2C12 myoblasts is highly dependent on the medium with which the cells interact. Thus, in the presence of differentiation medium, the cells changed. Initially, they adopted a fusiform or irregular triangle form, with 80% cell fusion. On the 2nd day, the cells displayed a plurinuclear aspect, while maintaining fibroblast-like features. On the 4th day, the inception of multinucleated myotubes was detected, as were the well-defined, mature C2C12 myotube cells on the 6th day (Supplementary Fig. S2).

### KPC-exosomes readily enter C2C12 myotubes

**Figure 1:**
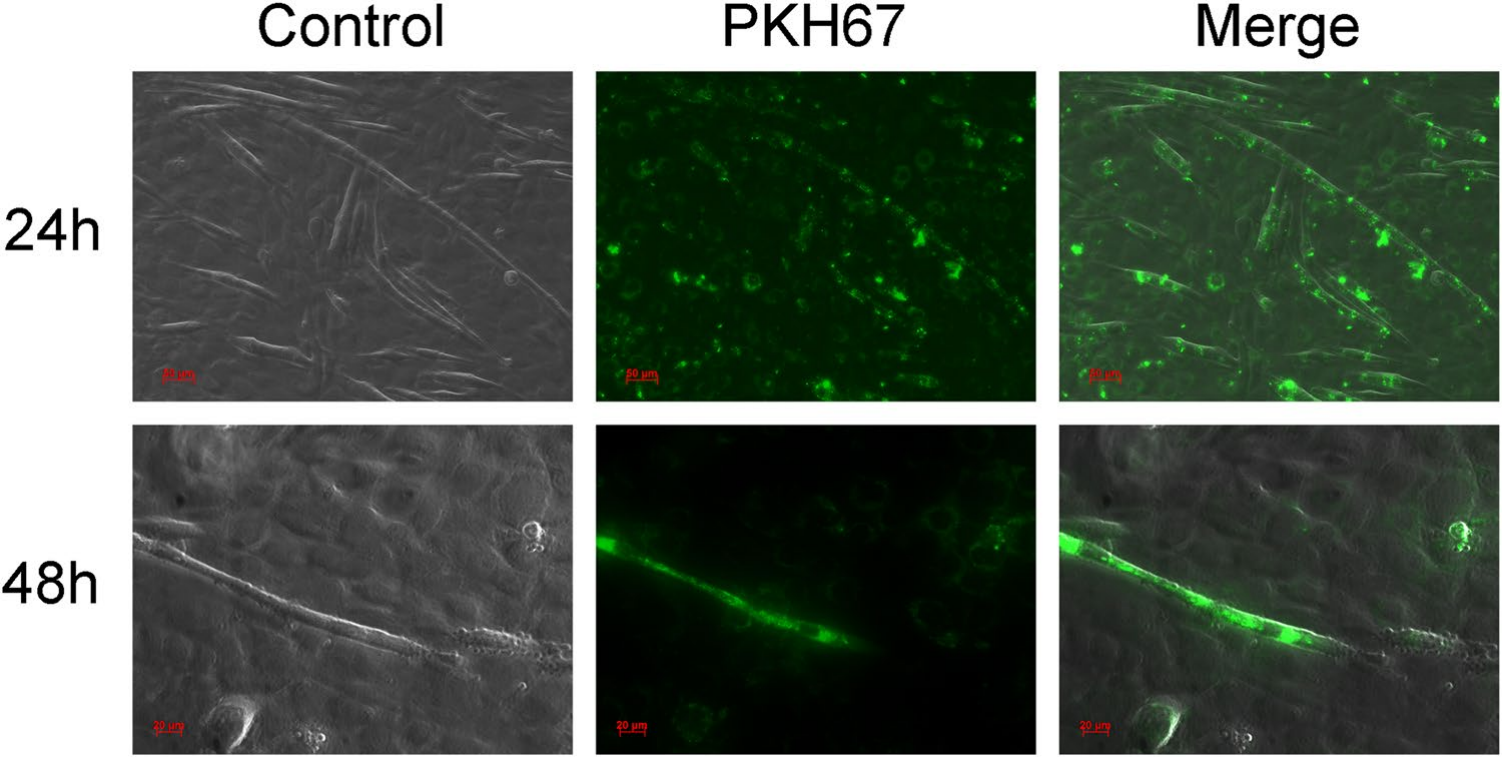
KPC-exosomes readily enter C2C12 cells. Fluorescence microscopy of differentiated C2C12 cells after co-incubation with 20 μg/ml PKH67-dyed KPC-exosomes (green) for 24 hours and 48 hours. Scale bar, 20 µm, 50 µm.

The differentiated C2C12 myotubes were incubated with 20 μg/mL PKH67-dyed exosomes isolated from the KPC cells. The exosomes incorporated into myotubes were identified after 24 h of co-incubation, using fluorescence microscopy (Carl Zeiss, Germany); 48 hours later, the green spots started diffusing (Fig. 1).

### The effect of KPC-exosomes on C2C12 cell survival and glucose uptake

**Figure 2:**
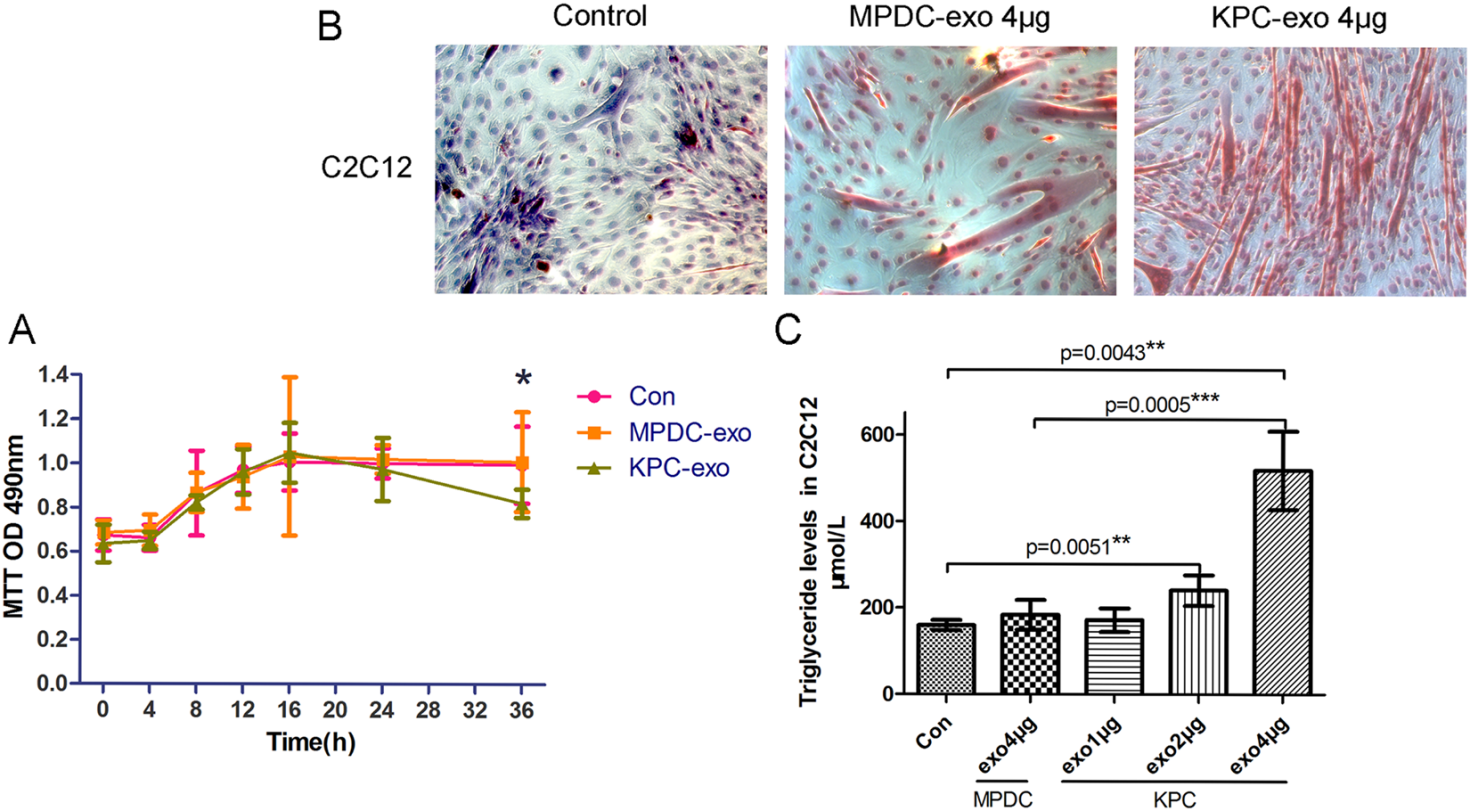
The effect of KPC-exosomes on the survival and triglyceride level of C2C12 cells. (**A**) Cell viability at different time points (0 h, 4 h, 8 h, 12 h, 16 h, 24 h and 36 h). *P < 0.05. Cells were cultured in 96-well plates treated with 5 μg/ml exosomes in each well. (**B**) Oil Red O Staining was used to observe lipidosis in C2C12 myotubes. Cells were cultured in 25-cm^2^ culture-flasks with 5 ml DMEM. Images were captured at 20× magnification. (**C**) The intracellular triglycerides of the C2C12 myotubes were quantified and analysed. Cells were cultured in 25-cm^2^ culture-flasks with 5 ml DMEM. *P < 0.05, **P < 0.01, ***P < 0.001.

We hypothesized that KPC-exosomes induced an inhibitory effect on the glucose uptake process of C2C12 cells. To determine whether cancer-derived microvesicles/exosomes with microRNAs could trigger apoptosis of skeletal muscle cells^20^, MTT colourimetric studies were performed to detect cell viability. The results indicated that KPC-exosomes exhibited minor effects on the survival behaviour of C2C12 cells over 24 hours (Fig. 2A) and that MPDC-exosomes minimally affected cell viability. However, glucose uptake was inhibited (Table 1).

**Table 1:** The optical density values and glucose concentrations of supernatants after incubations with KPC-exosomes for different times.

TIME	OD 490 nm		Glucose Concentration (mM)	
/h	Control	Kpc-exo 1 μg	Control	Kpc-exo 1 μg
2	0.042 ± 0.0017	0.049 ± 0.0053	0.50 ± 0.43	2.82 ± 1.75
4	0.042 ± 0.0008	0.054 ± 0.0074	0.32 ± 0.27	4.57 ± 2.76
6	0.048 ± 0.0036	0.069 ± 0.0112	2.24 ± 1.19	9.25 ± 3.75 **
16	0.046 ± 0.0013	0.056 ± 0.0053	1.59 ± 0.42	4.84 ± 1.77 *
24	0.044 ± 0.0041	0.054 ± 0.0041	1.24 ± 0.88	4.24 ± 2.86

The original glucose concentration of the supernatants was 15 mM. n = 4/each time point·group. Values are the MEAN ± SD. *p < 0.05 vs Control. **p < 0.01 vs Control.

### The effect of KPC-exosomes on the triglyceride level in C2C12 cells

According to previous studies, lipidosis within the skeletal muscle is closely related to insulin resistance^21, 22^. Our results were consistent with these studies, indicating that the triglyceride level in C2C12 cells treated with KPC-exosomes increased significantly in a dose-dependent manner. No substantial difference between the control group and the MPDC-exosomes-treated cells was observed (Fig. 2B and C).

### KPC-exosomes down-regulate the insulin and PI3K/Akt signalling pathways

Phosphatidylinositol 3-kinase (PI3K) is a heterodimeric lipid kinase containing catalytic (p110) and regulatory (p85) subunits, which are further divided into miscellaneous isoforms^23^. Protein kinase B (PKB, also known as Akt) plays a regulatory role in several essential cellular functions, being involved in tumour metastasis and tumourigenesis^24^. A cascade-like phenomenon occurs in which PTEN inhibits the activation of phosphorylated Akt, which subsequently induces the inhibition of the PI3K/Akt signalling pathway^25^.

**Figure 3:**
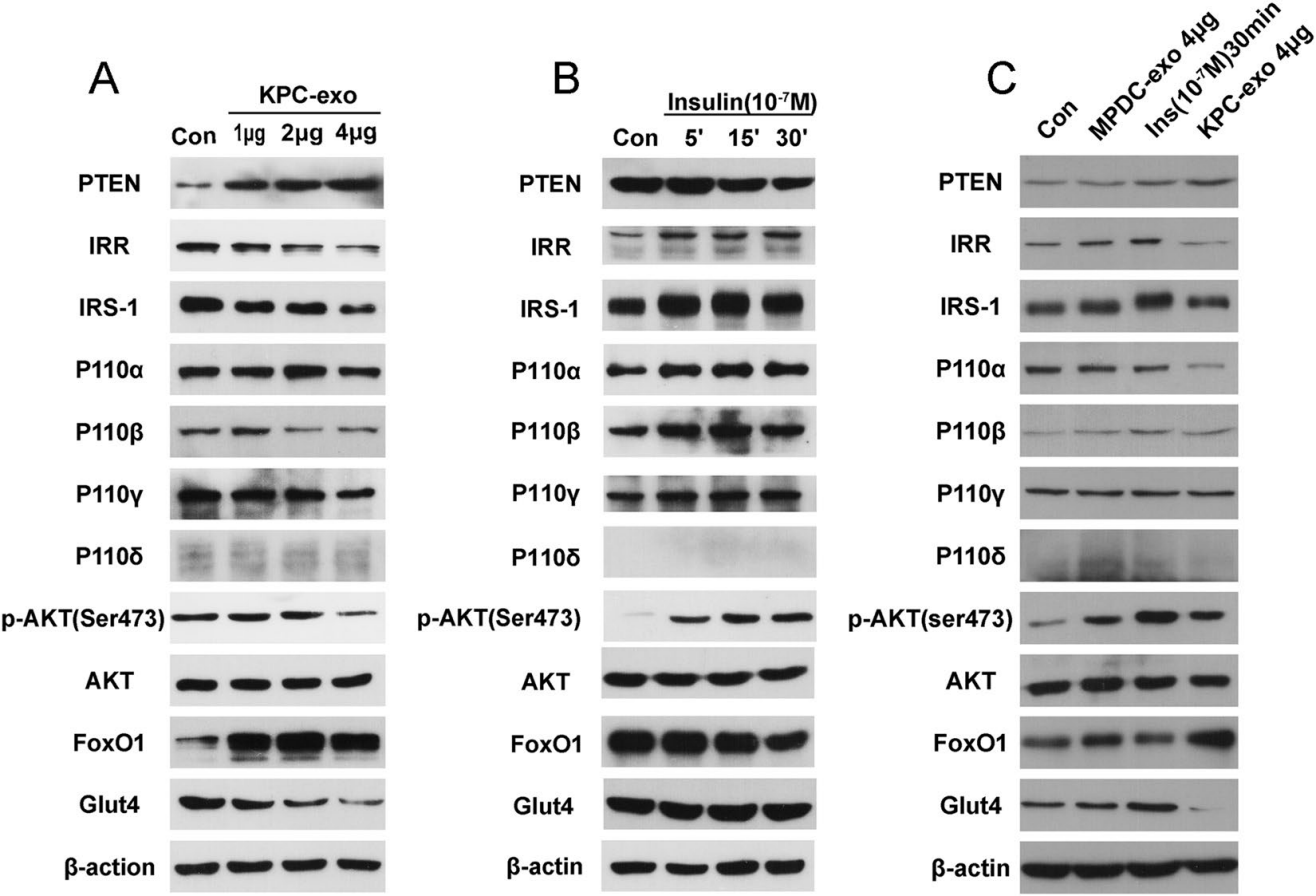
KPC-exosomes down-regulate the insulin and PI3K/Akt signalling pathway. To verify insulin and PI3K/Akt signalling pathway activation, the expression of some of the best characterized components and related targets was determined by western blot analysis. Differentiated C2C12 cells were cultured in 25-cm^2^ culture-flasks with 5 ml DMEM medium. (**A**) Differentiated C2C12 cells were treated with KPC-exosomes (1 μg, 2 μg, 4 μg) for 24 h. (**B**) Differentiated C2C12 cells were treated with insulin (10^−7^ M) for 5 min, 15 min, or 30 min. (**C**) Differentiated C2C12 cells were treated with 4 μg MPDC-exosomes for 24 h, 10^−7^ M insulin for 30 min, or 4 μg KPC-exosomes 24 h. MPDC-exosomes were employed as a negative control.

The detection of the expression of specific factors by western blot analysis —including PTEN, IRR, IRS-1, and p110α, β, γ, and δ, along with total and phosphorylated Akt (Ser473) and glucose transporter 4 protein (GLUT4) — has facilitated the investigation of PI3K/Akt signalling pathway and insulin activation. The results revealed that most of the above elements underwent down-regulation because of KPC-exosomes, except PTEN, which was up-regulated in a dose-dependent manner (Fig. 3A). To date, it is widely accepted that p110δ exists only in leucocytes, which is consistent with our results.

The physiological dose of insulin allows the translocation of GLUT4 from the intracellular medium into the plasma membrane, a process directly related to cellular glucose intake^26^. We observed that KPC-exosomes apparently depleted the expression level of GLUT4, as depicted in Fig. 3A. The western blot approach was a valuable tool in assessing the different functions played by insulin and KPC- and MPDC-exosomes (Fig. 3B and C).

We explored the existence of other extracellular vesicle subfractions, such as microvesicles known to act as carriers of cellular messages^27^, and showed that KPC-microvesicles had no impact on the protein level (Supplementary Fig. S3).

These results demonstrated that KPC-exosomes could inhibit insulin and the PI3K/Akt signalling pathway, presumably triggering down-regulation of glucose intake and subsequent insulin resistance. MPDC-exosomes were properly selected as the negative control in this study.

### FoxO1 and Phospho-FoxO1 expression and nuclear translocation are induced by KPC-exosomes in C2C12 myotubes

FoxO1 is a transcription-associated protein that is localized within the cell nucleus and is a key target of the PI3K/Akt signalling pathway. FoxO1 is phosphorylated by activated Akt, leading to nuclear exclusion and transcriptional activity suppression. Once activated, FoxO1 plays a critical role in muscle insulin resistance^28^.

**Figure 4:**
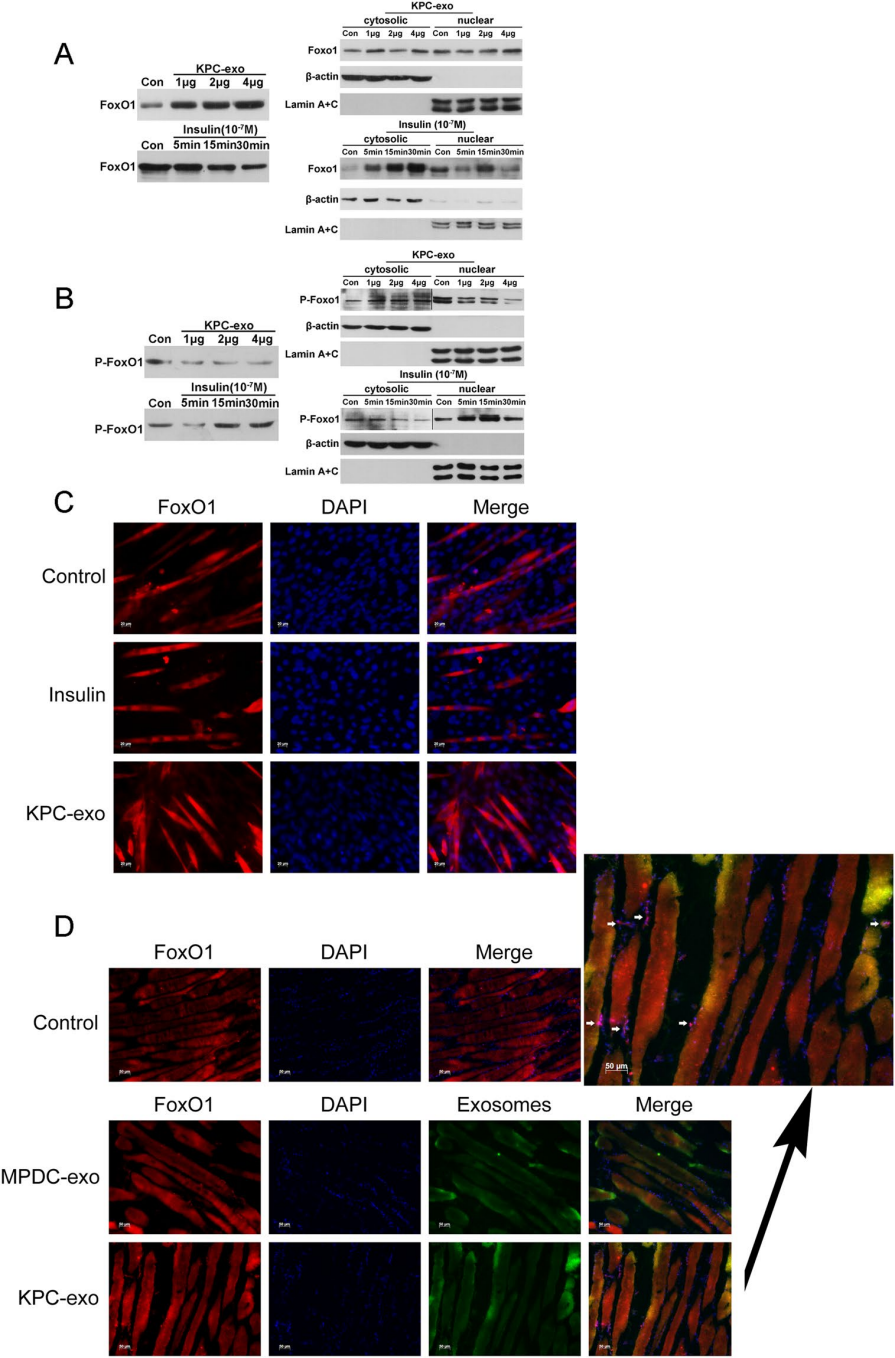
FoxO1 and Phospho-FoxO1 expression and nuclear translocation induced by KPC-exosomes in C2C12 myotubes. Differentiated C2C12 cells cultured in 25-cm^2^ culture-flasks with 5 ml DMEM were treated with KPC-exosomes (1 μg, 2 μg, 4 μg) for 24 h and 10^−7^ M insulin (5 min, 15 min, 30 min). (**A**) Total FoxO1 expression; expression in cytoplasm and nucleus. (**B**) Total Phospho-FoxO1 expression; expression in cytoplasm and nucleus. (**C**) Differentiated C2C12 cells cultured in 24-well plates were treated with 1 μg/ml KPC-exosomes for 24 h or 10^−7^ M insulin for 30 min. The nuclear (blue) translocation of FoxO1 (red) was verified by immunofluorescence. Scale bar, 20 μm. (**D**) 20 μg PKH67-dyed (green) exosomes in 100 μL PBS was injected through the caudal vein into the body of each mouse. The nuclear (blue) translocation of FoxO1 (red) was verified by immunofluorescence. Scale bar, 50 μm.

Our results showed that KPC-exosomes induced up-regulation of total FoxO1 in a dose-dependent manner. The nuclear FoxO1 levels displayed an increasing trend (normalized to the nuclear protein Lamin A/C levels; Fig. 4A) in contrast to the nuclear Phospho-FoxO1 levels (Fig. 4B). Compared to KPC-exosomes, insulin within physiological doses played an opposite role in FoxO1 nuclear translocation (Fig. 4A and B), which was confirmed by immunofluorescence. According to our *in vitro* studies, FoxO1 within the nucleus (red overlapped with blue) was mainly localized within the KPC-exosomes group rather than the insulin region (Fig. 4C), whereas *in vivo* analyses showed that the FoxO1 protein accumulated in the muscle cell nuclei (red overlapped with blue) of the nude mice (Fig. 4D).

### KPC-exosomes inhibit translocation of Glut4 to the plasma membrane

**Figure 5:**
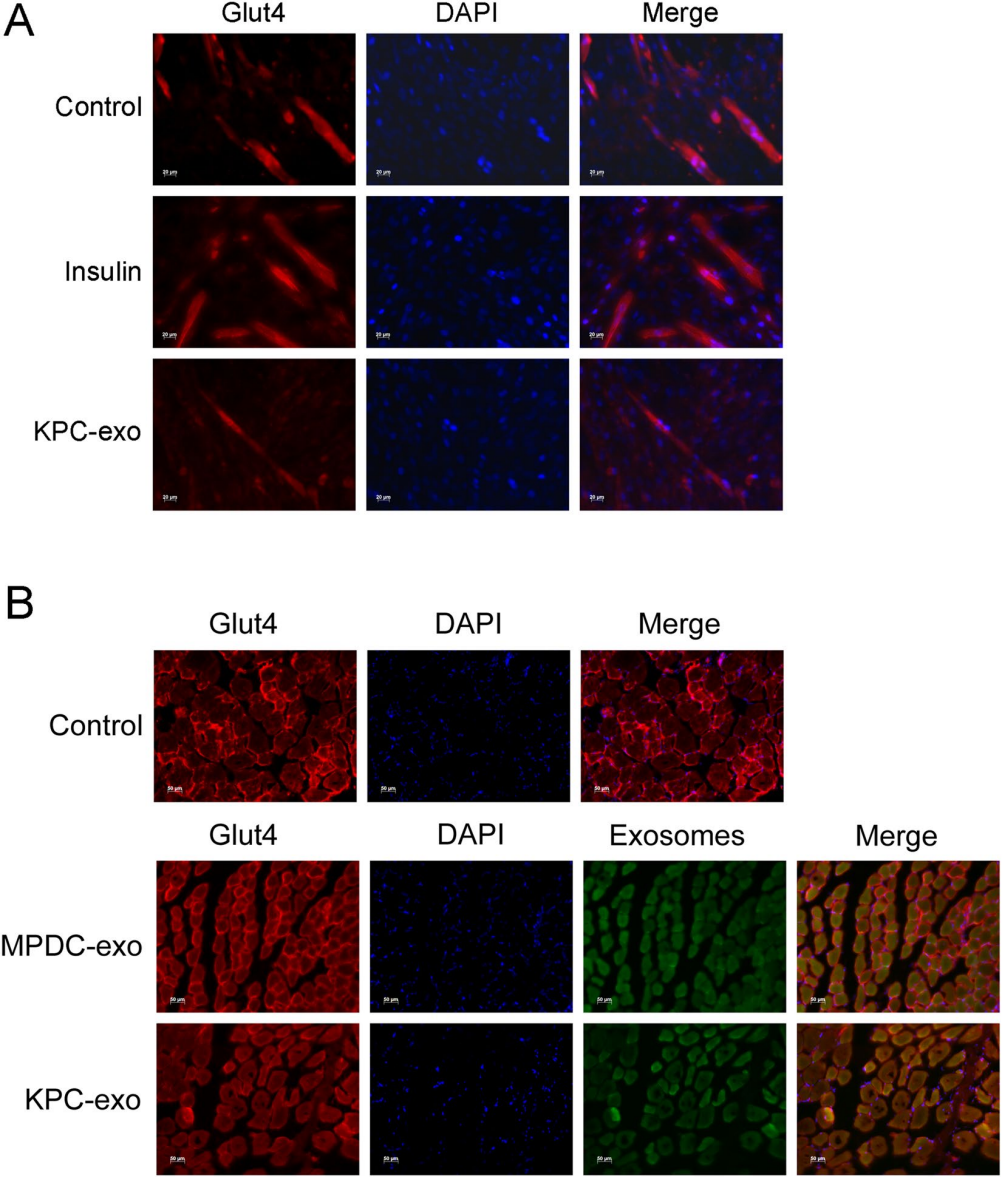
KPC-exosomes inhibit Glut4 translocation to the plasma membrane. (**A**) Differentiated C2C12 cells cultured in 24-well plates were treated with 1 μg/ml KPC-exosomes per well for 24 h or 10^−7^ M insulin for 30 min. The membrane distribution of Glut4 (red) was verified by immunofluorescence. Scale bar, 20 μm. (**B**) 20 μg PKH67-dyed (green) exosomes in 100 μL PBS was injected through the caudal vein into each mouse. The membrane distribution of Glut4 (red) was verified by immunofluorescence. Scale bar, 50 μm.

Regulation of the Glut4 distribution during glucose homeostasis maintenance is of paramount importance^26^. Under basal conditions, Glut4 exists mainly within the endomembranes of endosomes or certain intracellular vesicles. Once stimulated by insulin, Glut4 is transported to the plasma membrane by vesicles, promoting glucose uptake. To provide a comprehensive overview of Glut4 translocation governed by KPC-exosomes, we monitored the responses of these processes in C2C12 myotubes and skeletal muscle samples by immunofluorescence analysis. For this purpose, we employed and compared the KPC-exosomes and MPDC-exosomes groups together with a blank control group. The expression level of Glut4 within the membranes of the C2C12 myotubes was significantly decreased after interacting with KPC-exosomes compared with the blank control and MPDC-exosomes groups (Fig. 5A). Similarly, according to the *in vivo* experiments, KPC-exosomes induced an apparent depletion of the Glut4 distribution, as observed in Fig. 5B.

### FoxO1 depletion promotes Glut4 expression and minimizes the inhibition of Glut4 induced by KPC-exosomes *in vitro*

**Figure 6:**
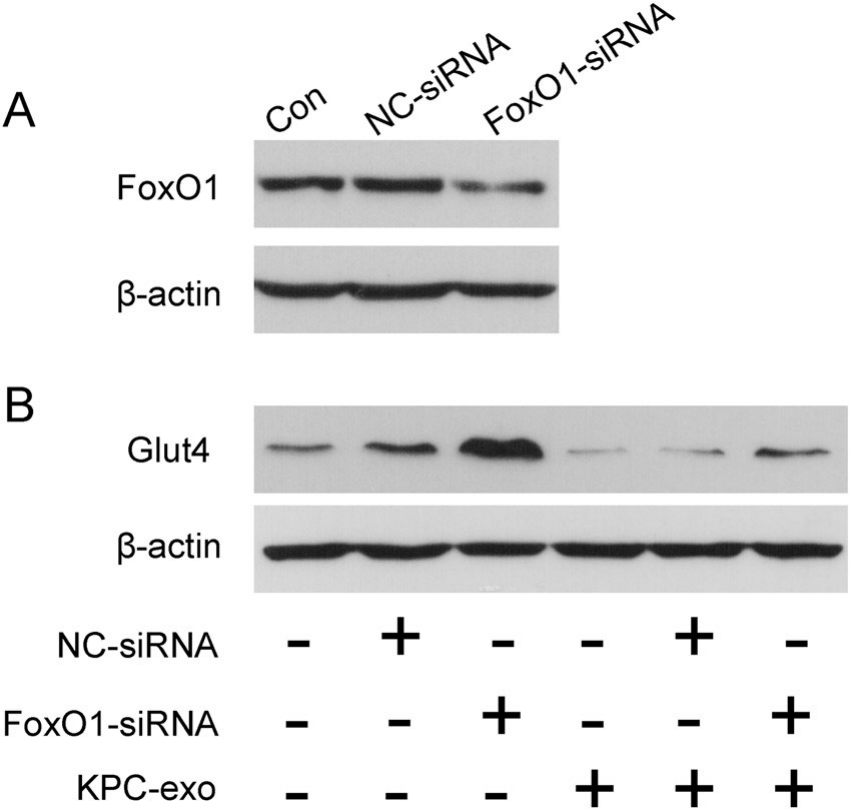
Effect of FoxO1 depletion on Glut4 expression. (**A**) The effect of siRNA on total FoxO1 protein expression. The total FoxO1 protein level was significantly decreased compared to the control groups. (**B**) The Glut4 protein level was dramatically enhanced when FoxO1 was depleted, which also minimized the inhibition of Glut4 induced by KPC-exosomes.

To investigate the effect of FoxO1 on Glut4, siRNA-FoxO1 was employed. After transfection, the total protein levels of FoxO1 were evaluated by western blot analysis, which showed a major loss (Fig. 6A). Subsequent results also suggested that FoxO1 depletion could promote Glut4 expression, while restraining the inhibition of Glut4 by KPC-exosomes (Fig. 6B). However, the underlying mechanism requires further exploration.

### MicroRNA microarray analysis and differential screening

**Figure 7:**
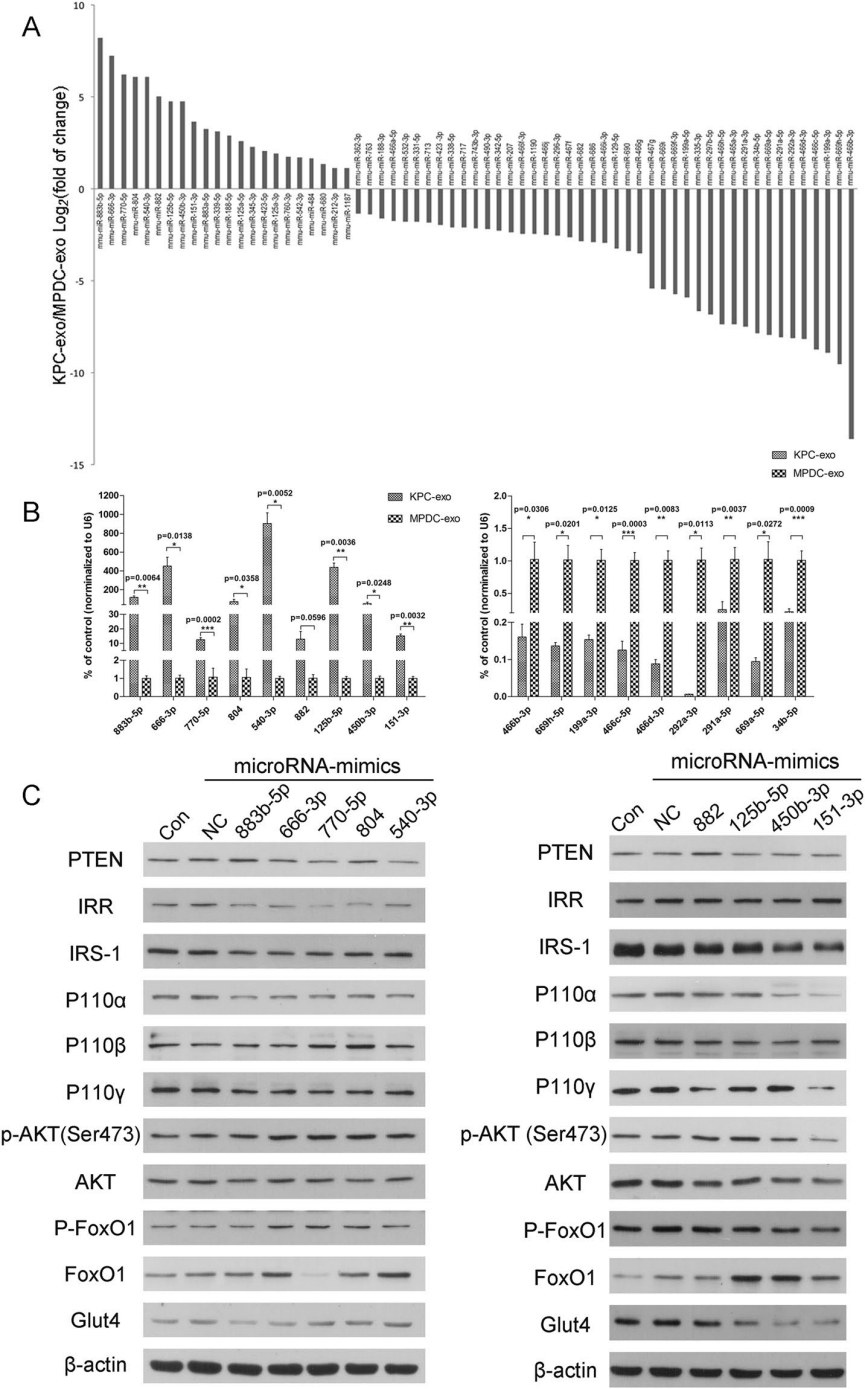
Comparison of miRNA profiles of the two types of exosomes according to the microarray analysis and validation by RT-qPCR and immunoblotting. (**A**) The bar graph shows differentially expressed miRNAs in KPC-exosomes compared with MPDC-exosomes. (**B**) The first nine miRNAs from each subgroup (increased & decreased) were validated by RT-qPCR. U6 was used as an endogenous control. (**C**) The effects of the first nine highly expressed miRNAs on the signalling pathways.

The expression profiles of exosomal microRNAs from KPC and MPDC cells were evaluated using the Agilent Mouse miRNA V21.0 chip (GSE95741). Compared with MPDC-exosomes (the negative control), a total number of 1,881 differentially expressed KPC-exosomal miRNAs were identified (Supplementary Information datasheet 1). Using specific filter criteria, where one or both of the KPC- and MPDC-exosomes exhibited a distinct signal against the microarray background (marked as “Detected” in datasheets) and a ≥ two-fold change in expression was evident, 799 differentially expressed miRNAs were selected (Supplementary Information datasheet 2), of which 339 were up-regulated, and 460 were down-regulated (Supplementary Information datasheet 3). We initially focused on the insulin, PI3K/Akt, and FoxO pathways (data provided in Supplementary Information datasheet 4). Among these signalling pathways, 65 differentially expressed miRNAs were identified (22 highly expressed and 43 lowly expressed; Fig. 7A and Supplementary Information, datasheet 5). Validation of the microarray data was performed by RT-qPCR analyses, using the first nine miRNAs from each subgroup (high and low expression; Fig. 7B). We also explored the effects on the signalling pathways of the first nine highly expressed miRNAs and discovered that miR-666-3p, miR-540-3p, miR-125b-5p and miR-450b-3p potentially promote FoxO1 expression, whereas miR-883b-5p, 666-3p, miR-450b-3p and miR-151-3p may play an essential role in down-regulation of Glut4 expression. Considering the expression changes in the upstream proteins in the IRS/PI3K/Akt signalling axis, the regulation of FoxO1 and Glut4 presumably occurs in an indirect manner (Fig. 7C).

### Target gene prediction and GO/KEGG pathway enrichment

To identify the potential target genes of the differentially expressed miRNAs, gene prediction studies were performed using three established algorithms (TargetScan, PITA and microRNA.org), with a total number of 12301 putative targets being identified (Supplementary Information datasheet 6). For a comprehensive understanding of the functions of the predicted genes, the targets were subjected to a Gene Oncology (GO) analysis, employing three categories: cellular component, molecular function and biological process. The membrane, cytoplasm, nucleus, protein binding and regulation of transcription categories were classified as highly enriched categories that were mainly involved in cellular material transportation, signal transduction and homeostatic processes (Supplementary Fig. S4). Additionally, these predicted genes were classified according to KEGG functional annotations to identify the actively regulated pathways. The results listed 281 relevant signalling pathways (Supplementary Information datasheet 4), among which the PI3K/Akt, FoxO, and insulin signalling pathways were markedly enriched (Table 2 and Supplementary Information datasheet 4). We assume that these signalling pathways are promising platforms for further investigations of underlying mechanisms, and we have designed a brief scheme showing the complex correlation behind these processes (Supplementary Fig. S5).

**Table 2:** KEGG pathways enriched by target genes of differentially expressed miRNAs.

NO.	Pathway ID	Pathway description	Target genes with pathway annotation, n (%)	P value
1	mmu05200	Pathways in cancer	204(9.28)	4.09E-12
……		……	……	
5	mmu04151	PI3K-Akt signalling pathway	171(7.78)	5.44E-09
……		……	……	
14	mmu04120	Ubiquitin mediated proteolysis	78(3.55)	3.82E-06
15	mmu04068	FoxO signalling pathway	73(3.32)	8.01E-06
……		……	……	
23	mmu04910	Insulin signalling pathway	71(3.23)	8.70E-05
……		……	……	
190	mmu04140	Regulation of autophagy	10(0.45)	0.691436
……		……	……	

P < 0.05 with significant enrichment.

The likelihood of autophagy in this field was also considered, but the western analysis (Supplementary Fig. S6) revealed that the expression of microtubule-associated protein 1 light-chain 3 (LC3) — an important molecular indicator of autophagy activity — did not display a remarkable change in the LC3II/LC3I ratio; the KEGG pathway enrichments (Table 2 and Supplementary Information datasheet 4) did not support this possibility either.

## Discussion

Epidemiological prospective studies^5–8^ as well as frontier researches^29^ have clearly shown a connection between new-onset diabetes and PC, under which the novel cancer-related concepts “metabolic reprogramming” and “metabolic crosstalk”^10, 30, 31^ have gained increased attention. However, the previously reported studies do not fully justify the mechanism behind such metabolic crosstalk between PC and peripheral tissues. The current study not only comes to support these concepts but also contributes to the mechanism of the glucose metabolic disorder and insulin resistance within peripheral tissues in the context of PC.

IR of skeletal muscle is not only the main pathological component of PC-DM^9^ but it also has a close connection with the progression of PC^12, 13^. Therefore, when the mechanism of skeletal muscle IR induced by PC is clarified, we will identify some key molecules in the process, and the results may contribute to improvements in the diagnosis and treatment of PC.

We report herein that C2C12 cells treated with KPC-exosomes have induced insulin and PI3K/Akt signalling inhibition, triggering insulin resistance and glucose intolerance. We have detected increased levels of FoxO1 expression and nuclear translocation both *in vitro* and *vivo*. By contrast, Glut4 exhibited depleted expression, particularly within the cell membrane. Screening studies (microarray analysis) have offered a plethora of differentially expressed KPC-exosomal miRNAs, several of which are promising agents in mediating insulin signalling. According to these data, exosomal miRNAs may act as initiators of the PC-derived exosomal ability to disrupt the metabolic balance of skeletal muscles. These novel findings indicate that PC-derived exosomes may play a key role in the onset of PC-DM, and exosomal miRNAs are promising biomarkers in this field.

**Figure 8:**
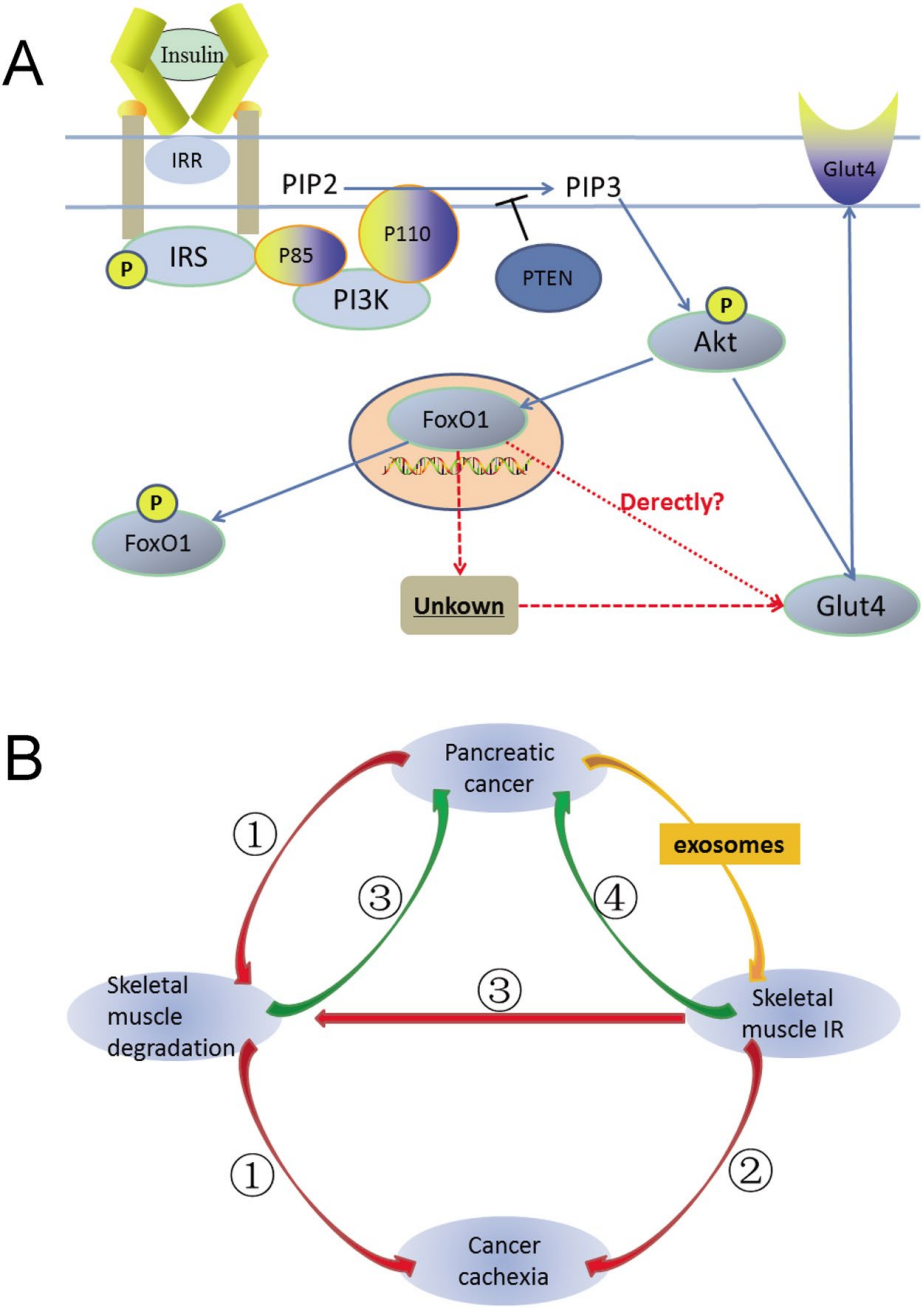
Schematic of the mechanism and magnificent background involved in this study. (**A**) Schematic of the insulin and PI3K/Akt signalling pathways. Dashed lines indicate a possible mechanism. (**B**) Schematic of the metabolic crosstalk between PC and skeletal muscle: ① UPS plays a key role in cancer patients’ experience of skeletal muscle loss; ② A close connection between the degree of severity of IR and progression of cancer cachexia; ③ IR exacerbates skeletal muscle degradation as cancer evolves, which produces amino acids for tumour proliferation; ④ Under the conditions of IR, overexpression of IRR and IGF-1 receptor in tumour cells promotes tumour growth.

The dysfunction of insulin signal transduction triggers insulin resistance; once this signal is weakened or disrupted, insulin resistance occurs. Insulin exerts biological functions by binding to specific receptors on the surfaces of insulin target cells^32^. Insulin activates tyrosine kinase activity through insulin receptor-related receptor (IRR), inducing insulin receptor substrate (IRS) phosphorylation. Phosphorylated IRS combines further with P85, which is the regulatory subunit of phosphatidylinositol 3-kinase (PI3K), leading to the activation of the catalytic subunit P110. Once activated, P110 catalyses the conversion of phosphatidylinositol-4,5-bisphosphate (PIP2) to phosphatidylinositol-1,4,5-trisphosphate (PIP3). PIP3 further activates proteins with the pleckstrin homology (PH) domain, promoting their membrane translocation. One of the proteins that contains a PH domain is a serine-threonine kinase (Akt), also known as protein kinase B (PKB), that represents the main signalling component of insulin signalling transduction. Therefore, the IRS/PI3K/Akt axis as an insulin signal transduction pathway is of paramount importance in mediating the subsequent series of insulin biological effects^33^. The transcription factor FoxO1 is expressed in various species and belongs to a subfamily of the Forkhead protein family, class O. FoxO1, the first discovered member of this subfamily, is a crucial molecular downstream regulator of the insulin signalling pathway, playing a key role in regulating glucose homeostasis^34^. Once phosphorylated by activated Akt, FoxO1 translocates from the nucleus to the cytoplasm, losing its transcriptional activity. However, when PI3K/Akt signalling is not activated, phosphorylated FoxO1 is dephosphorylated, re-translocated to the nucleus, where it recovers its transcriptional activity. Therefore, a physiological dose of insulin inhibits FoxO1 activity through activation of the PI3K/Akt signalling pathway, as illustrated in Fig. 8A.

Our results have clearly demonstrated that KPC-exosomes negatively regulate the insulin and PI3K/Akt signalling pathway (Fig. 3A), in contrast to the effect of a physiological dose of insulin (Fig. 3B). According to these data, we speculate that exosomal miRNAs play a critical role in this process (Fig. 7C and Supplementary Fig. S3). It is worth mentioning that one of the predicted genes of miR-151-3p is Ppp2r5b (protein phosphatase 2, regulatory subunit B’, beta isoform) (Supplementary Information datasheet 6), which has been linked to insulin resistance in adipocytes^35^. Although this study supports our assumptions from a different perspective, the specific mechanism of these exosomal miRNAs *in vitro* and *in vivo*, either through direct binding or by indirectly regulating the expression of other genes, requires further investigations.

FoxO1 and Glut4 are closely linked to insulin resistance and glucose intake, respectively (Fig. 8A). Thus, we have explored their expression and distribution. According to our immunoblotting analyses, KPC-exosomes facilitated FoxO1 protein expression but inhibited Glut4, as shown in Fig. 3A. Subsequent studies indicated an increased level of FoxO1 nuclear translocation that was induced by KPC-exosomes, while Phospho-FoxO1 showed a decreasing trend (Fig. 4A and B). We further validated the translocation of FoxO1 and Glut4 *in vitro* (Fig. 4C and A) and *in vivo* (Figs 4D and 5B) by immunofluorescence.

FoxO1 and Glut4 are the most essential downstream targets of PI3K/Akt signalling. It has been reported that FoxO1 can inhibit the transcription of peroxisome proliferator-activated receptors PPAR-γ1 and PPAR-γ2 in adipocytes, thereby circumventing the inhibition of PPAR-γ2 on Glut4^36^. However, the potential existence of a regulatory relationship between FoxO1 and Glut4 within skeletal muscles is questionable. We transfected C2C12 cells with siRNA-FoxO1 and showed that the FoxO1 defect can promote Glut4 expression and partially restraining the inhibition of KPC-exosomes on Glut4 (Fig. 6B). This valuable discovery paves the way for further studies to elucidate it (Fig. 8A).

The microRNA microarray (GSE 95741) and KEGG analyses have confirmed the appropriate selection of insulin, PI3K/Akt and FoxO as signalling pathways in this study (Table 2 and Supplementary Information datasheet 4). Based on the essential impact of autophagy on metabolism^37^, we also attempted to explore potential correlations of autophagy with this field. To this end, we chose the microtubule-associated protein 1 light-chain 3 (LC3) type I and II, a homologue of the yeast ATG6 (Ant7/Apg8) gene in mammalian cells. When autophagy occurs, cytoplasmic LC3 cleaves small segments of polypeptides through enzymolysis and becomes LC3-I. LC3-I combines further with phosphatidyl ethanolamine (PE) and undergoes lipidation by a ubiquitin-like system to generate LC3-II, which binds to the autophagic membrane^38^. Therefore, the amount of LC3-II (LC3II/I ratio) is correlated with the autophagy level, being a reliable marker for monitoring the autophagic course. However, our results (Supplementary Fig. S6) indicated no apparent difference in LC3II/I, combined with the KEGG data (Table 2), suggesting that autophagy is probably not involved in these processes.

The KEGG results have also indicated that the ubiquitin-proteasome pathway (or ubiquitin-proteasome system, UPS) is enriched by the predicted target genes of differentially expressed KPC-exosomal microRNAs. This is of paramount importance as it highlights essential features (Fig. 8B). First, UPS plays a key role in cancer patients experiencing skeletal muscle loss; its excessive loss is a fundamental feature of cancer cachexia^39^. In addition, a close connection between the degree of severity of IR and progression of cancer cachexia has been observed^12, 13^; IR exacerbates skeletal muscle degradation as cancer evolves, and the amino acids produced by muscle breakdown are used for tumour proliferation^30^. Lastly, under the conditions of IR, the serum insulin level increases, leading to the overexpression of IRR and the insulin-like growth factor 1 (IGF-1) receptor in tumour cells, which promotes tumour growth^40, 41^. In the context of PC “metabolic reprogramming” and “metabolic crosstalk”, we can evaluate this finding from a macroscopic perspective.

In summary, we have demonstrated that PC-derived exosomes (isolated from murine pancreatic cancer cells) inhibit glucose intake and promote lipidosis, developing an eventual state of insulin resistance in skeletal muscle cells. The process is, at least partially, governed by the PI3K/Akt/FoxO1 signalling pathways, and exosomal miRNAs are probably involved. The current study provides new insight into the mechanism of PC-DM and will contribute to a comprehensive understanding of the relationship between PC and systemic metabolism. Subsequent studies will explore the potential of promising exosomal miRNAs and include functional verifications and mechanistic elucidations. This study paves the way towards discovering potential targets for correcting metabolic disorders and improving the diagnosis/treatment of PC.

## Materials and Methods

### Cell culture

The murine pancreatic cancer cell line, developed from the tumour tissues of *Kras* ^*LSL-G12D/*+^, *Trp53* ^*LSL-R172H/*+^, *Pdx1-Cre*(*KPC*) mice that can develop spontaneous pancreatic ductal adenocarcinoma (PDAC), was kindly provided by Dr. Tingbo Liang’s Research Group (Zhejiang University, China) and maintained in RPMI-1640 medium (Gibco, Shanghai, China), supplemented with 20% foetal bovine serum (FBS), 1% sodium pyruvate (100×), and 1% MEM Non-Essential Amino Acids (NEAA 100×). The murine pancreatic ductal epithelial cell line (MPDC) was purchased from CELLBIO Company (CBR131654, Shanghai, China) and cultured in Dulbecco’s modified Eagle’s Medium containing L-Glutamine, 4.5 g/L D-Glucose, and 110 mg/L sodium pyruvate (DMEM; Gibco, Shanghai, China) and supplemented with 10% FBS. C2C12 myoblasts were purchased from the cell bank of the Chinese academy of sciences (GNM26, Shanghai, China) and cultured in DMEM, supplemented with 10% FBS. When C2C12 myoblasts achieved 70–80% confluence, the medium was replaced with DMEM containing 2% horse serum daily, which would induce the differentiation of myoblasts into myotubes in 4–6 days. The myotubes were used for the experiments. The images of the C2C12 differentiation process were obtained by fluorescence microscopy (40×, Carl Zeiss, Germany) in coordination with an imaging system (Ckx41, Olympus).

### Exosome isolation

Exosomes, secreted by the murine pancreatic cancer cells (KPC-exosomes) and the murine pancreatic ductal epithelial cells (MPDC-exosomes), were isolated from supernatants. Cells were cultured to 80% confluence, and the medium was replaced with medium containing exosome-free FBS (prepared by differential centrifugation for 16 h at 100,000 g). Supernatants were collected after 48 h of incubation. Exosomes were then isolated by differential centrifugation as previously described^42^. It is worth mentioning that after the step involving 10,000 g for 30 minutes, the pellet also included microvesicles, which are large EVs ranging from 50 to 1,000 nm in diameter^42^. The final exosome pellet was resuspended in 1 × PBS for use.

### qNano analysis

Isolated exosomes were analysed on the qNano-TRPS (Izon Science Ltd, China). Fractions were diluted and 5-minute movies were generated for 40 μl of each sample. Graphical analysis showed the concentration per millilitre and the particle size distribution of the microparticles.

### Electron microscopic imaging of exosomes

A drop of 20 μl of the exosome suspension (1–2 μg/μl) was pipetted onto an electron-microscopy grid and allowed to stand for 3–5 minutes at room temperature. Then, the grid was moved to the surface of a drop of 1% osmium tetroxide (diluted in 0.1 M sodium phosphate buffer) for another 3–5 minutes to be stained. Excess fluid was removed with a piece of Whatman filter paper. Exosomes were viewed using the Philips Tecnai 10 transmission electron microscope (Philips, Netherlands).

### Western blot of exosomes

Exosomes were isolated according to the procedure mentioned above. The exosome suspension was disintegrated with RIPA Lysis Buffer (Sigma, USA) containing a protease inhibitor cocktail kit (Thermo, USA) for 10 minutes at room temperature and quantified using the bicinchoninic acid assay (Pierce, USA). Samples were prepared for western blot according to standard protocols, and the PVDF membranes were probed with the following antibodies: Alix (#2171, Cell Signalling Technology/CST), CD63 (ab68418, Abcam) and Calnexin (sc-23954, San Cruz Biotechnology).

### Exosome internalization and fluorescence microscopy analysis

Exosomes were isolated from KPC cells and dyed with green fluorescent linker PKH67 (#SLBN9941V, Sigma) according to the manufacturer’s protocol; 20 μg/ml of PKH67-dyed exosomes were used to treat differentiated C2C12 myotubes in a 15-mm culture dish. Fluorescence microscopy (Carl Zeiss, Germany) images were obtained after 24 and 48 hours.

### Cell viability assay

Differentiated C2C12 myotubes cultured in 96-well plates were divided into 3 groups (28 wells/group) and treated with 5 μg/ml KPC-exosomes, 5 μg/ml MPDC-exosomes or a blank control.

After 0 h, 4 h, 8 h, 12 h, 16 h, 20 h and 24 h of incubation (4 wells/each time point·group), cell viability was determined using the 3-(4,5-dimethyl-2-thiazolyl)-2,5-diphenyl-2H-tetrazolium bromide (MTT) assay (Sigma). Cells were incubated with 20 μl MTT for 4 h, the supernatant was removed and 150 μl DMSO was added to each well. After 10 min of shaking, the optical density value was measured by an ELISA reader (BioTek ELx800, Winooski, VT, USA) at 490 nm. The curve was plotted with the optical density value as the y-axis and the time interval as the x-axis.

### Oil red O staining and triglyceride assay

Differentiated C2C12 myotubes cultured in 25-cm^2^ culture-flasks (with 5 ml DMEM) were divided into 3 groups treated with 4 μg KPC-exosomes, 4 μg MPDC-exosomes or a blank control. After 24 h of incubation, an Oil Red O Stain Kit (D207, Jancheng Bioengineering Institute, Nanjing, China) was used to detect lipidosis in C2C12 myotubes. Images were captured at 20× magnification using an OLYMPUS CKX41 imaging system incorporated into the microscope (Carl Zeiss Microscopy GmbH. US.). Intracellular triglycerides were detected using a triglyceride assay kit (E1013, Applygen Technologies Inc. Beijing, China).

### Glucose uptake assay

Differentiated C2C12 myotubes cultured in 24-well plates divided into 2 groups (treatment & control, 20 wells/group) were serum starved for 2 h and incubated with 1 μg/ml KPC-exosomes at 37 °C in serum free media for 2 h, 4 h, 6 h, 16 h and 24 h (4 wells/each time point·group). Afterwards, insulin (I6634, Sigma) was added to each well to 10^−7^ M for another 10 minutes at 37 °C. Cells were washed twice with Kreb’s buffer (136 mM NaCl, 4.7 mM KCl, 1.25 mM MgSO4, 1.2 mM CaCl2, 20 mM HEPES, pH 7.4), which was kept at room temperature prior to use. Afterwards, 850 μl Kreb’s buffer with 150 μl D-(+)-Glucose (VETEC) solution (100 mM) was added to each well for the last 10 minutes at 37 °C. The glucose concentration of the supernatant in each well was detected with the glucose oxidase kit (E1010, Applygen Company, China).

### Western blot analysis of cell-based experiments

Differentiated C2C12 myotubes grown in 25-cm^2^ culture-flasks (containing 5 ml DMEM) were treated with KPC-exosomes (0 μg, 1 μg, 2 μg, 4 μg), MPDC-exosomes (4 μg) and insulin (10^−7^ M: 0 min, 5 min, 15 min, 30 min).

Procedures for protein extractions and immunoblots were carried out according to standard protocols. Nuclear and cytoplasmic proteins were extracted using the NE-PER Nuclear and Cytoplasmic Extraction Reagents (78833, Thermo Scientific, Waltham, MA, USA). Primary antibodies against PTEN (ab32199, Abcam), IRR (ab40782, Abcam), IRS-1 (#3407, CST), PI3 Kinase p110α (ab40776, Abcam), PI3 Kinase p110β (ab151549, Abcam), PI3 Kinase p110γ (#5405, CST), PI3 Kinase p110δ (ab109006, Abcam), Phospho-Akt (Ser473) (#4060, CST), Akt (#2920, CST), FOXO1 (#2880,CST), Phospho-FoxO1(Ser256)(#9461, CST), Glut4 (#2213, CST), Lamin A + C (ab108922, Abcam), β-actin (sc-47778, Santa Cruz Biotechnology), and LC3B (ab192890, Abcam) were used.

### Animal study

Six- to seven-week-old female athymic nude mice purchased from the Zhejiang Chinese Medical University (Zhejiang, China) were divided into three groups randomly (Control, KPC-exosomes, and MPDC-exosomes). Twenty micrograms of PKH67-dyed exosomes in 100 μL PBS was injected through the caudal vein into the body of each mouse. Forty-eight hours later, all mice were sacrificed by neck dislocation. Skeletal muscle samples were processed into frozen sections for immunofluorescence analysis. All animal experiments were approved by the Animal Experimentation Committee of Zhejiang University. All experiments were performed in accordance with relevant guidelines and regulations.

### Immunofluorescence analysis

Differentiated C2C12 cells cultured in 24-well plates were treated with 1 μg/ml KPC-exosomes for 24 h or 10^−7^ M insulin for 30 min. Cells were fixed with 4% paraformaldehyde for 15 minutes at 4 °C, blocked with 5% BSA for 1 h at 37 °C, and permeabilized with a PBS solution containing 5% Triton X-100 for 30 min at room temperature. Then, cells were incubated with a primary antibody against FOXO1 (#2880, CST) at 4 °C overnight, followed by another 1 h at room temperature and with Cy3-conjugated secondary antibody (GB21303, Goodbio technology Company, China). Nuclei were stained with DAPI (Sigma). Images were acquired using a confocal microscope (Zeiss, Jena, Germany). When the primary antibody was Glut4 (sc-53566, Santa Cruz Biotechnology), cells did not require permeabilization. For the frozen skeletal muscle sections, the permeation step was also omitted.

### RNA silencing by siRNA

Murine FoxO1 siRNA (5′-GCAGCAGACACCAUGCUAUTT-3′) and negative control (NC) were synthesized by GenePharma (Shanghai, China). After 3 days of differentiation, the C2C12 cells (grown in 6-well plates) were used for transfection. The FoxO1 siRNA (150 nM) and negative control (NC) (150 nM) were transfected into cells in antibiotic- and serum-free medium using Lipofectamine 2000 (Invitrogen, USA), according to the manufacturer’s instructions. Cells without transfection were used as the control group. Cells were collected at 72 h post-transfection (the C2C12 cells were incubated with exosomes in the last 24 h), and Glut4 protein expression was detected. Procedures for protein extractions and immunoblots were carried out according to standard protocols.

### MicroRNA microarray assay

MPDC-exosomes were used as a negative control. Through the miRNA microarray analysis, the differentially expressed miRNAs for KPC-exosomes were screened out. The microRNA microarray analysis was performed by OE Biotech. Co., Ltd. (Shanghai, China; http://www.oebiotech.com/). The Cy3-labeled RNA was purified before hybridization on an Agilent Mouse miRNA V21.0 chip (ID: 070155). Data analysis was initiated by first subtracting the background and then normalizing the signals using the GeneSpring software (version 13.1, Agilent). The threshold of significance was defined by the P value, with P ≤ 0.05 regarded as significant for the GO and KEGG analyses.

The validation of the microarray data was performed by RT-qPCR, using the StepOnePlus Real-Time PCR System (ABI, USA). The universal reverse primer was provided by TIANGEN BIOTECH CO. (Beijing, China). All other primer sequences are shown in Supplementary Table S1. The differences in the expression of miRNAs between KPC-exosomes and MPDC-exosomes were analysed using the ΔΔCT method and normalized to U6 expression^43^.

### Cell transfection

According to the microRNA microarray analysis, the first nine highly expressed miRNAs (miR-883b-5p, miR-666-3p, miR-770-5p, miR-804, miR-540-3p, miR-882, miR-125b-5p, miR-450b-3p, and miR-151-3p) were selected out for further investigation. The corresponding miRNAs mimics (150 nM) and negative control (NC) (150 nM) were synthesized by GenePharma (Shanghai, China). The remaining steps were the same as described above for RNA silencing by siRNA.

### Target gene prediction of differentially expressed microRNAs

Three computational prediction algorithms (TargetScan, PITA and microRNA.org) were used to predict the targets of the significantly changed miRNAs identified through the microarray analysis. The default parameters of the 3 databases were defined by the context score (%), which were set as >50%, 25%, and 25%, respectively, for TargetScan, PITA and microRNA.org. The shared target genes between the 3 databases were used for analysis.

### GO enrichment and KEGG pathway analysis of target genes

To comprehensively characterize the properties of the targets, the putative genes were subjected to GO enrichment and KEGG pathway analysis based on GENE2GO of NCBI and the KEGG database. The Fisher’s exact test was used to select the significant GO categories and signalling pathways. The threshold of significance was defined by the P value, with P ≤ 0.05 regarded as significant for the GO and KEGG analysis.

### Statistical analysis

Results are expressed as the means ± standard error. Analyses between different groups were performed using the two-tailed Student t-test or one-way analysis of variance. Statistical significance was set as follows: a P value of 0.05 was significant, and a value of 0.01 was highly significant.

### Data availability

All data generated or analysed during this study are included in this published article (and its Supplementary Information files).

## Electronic supplementary material

Supplementary information

Dataset 1

Dataset 2

Dataset 3

Dataset 4

Dataset 5

Dataset 6
