# Supplementary material for: Exosomes derived from pancreatic cancer cells induce insulin resistance in C2C12 myotube cells through the PI3K/Akt/FoxO1 pathway
Source: Sci Rep. 2017 Jul 14;7:5384. doi: 10.1038/s41598-017-05541-4 (PMC5511275; doi:10.1038/s41598-017-05541-4)
Supplement: Supplementary file 1 — Supplementary information [file 41598_2017_5541_MOESM1_ESM.pdf]

Supplementary

Title

Exosomes derived from pancreatic cancer cells induce insulin resistance in C2C12 myotube cells through the PI3K/Akt/FoxO1 pathway

Lantian Wang[1,2*], Bo Zhang[1,2*], Wen Zheng[1,2*], Muxing Kang[1,2], Qing Chen[1,2], Wenjie Qin[1,2], Chao Li[1,2], Yuefeng Zhang[1,2], Yingkuan Shao[2], Yulian Wu[1,2]

1 Department of Surgery, The Second Affiliated Hospital, Zhejiang University School of Medicine, Hangzhou, P. R. China

2 Key Laboratory of Cancer Prevention and Intervention, China National Ministry of Education, Cancer Institute, Second Affiliated Hospital, Zhejiang University School of Medicine, Hangzhou, Zhejiang, P. R. China

* These authors have contributed equally to this work

Correspondence to: Yulian Wu, M.D., Ph. D. Department of Surgery, Second Affiliated Hospital, Zhejiang University School of Medicine, 88 Jiefang Road, Hangzhou, 310009, China.
Tel.: +86 571 87784604 fax: +86 571 87784604
E-mail: wuyulian@medmail.com.cn

Figure S1. Identification of KPC-exosomes and MPDC-exosomes.

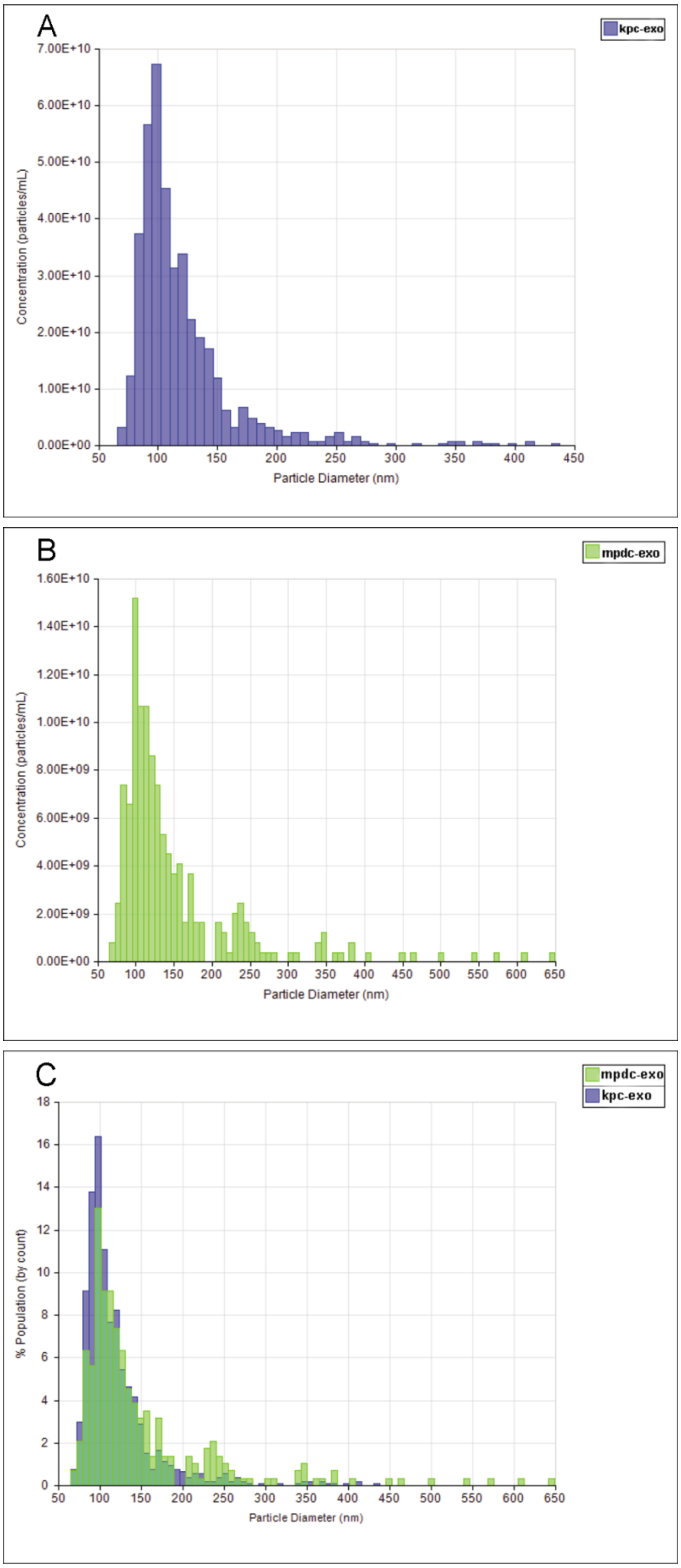

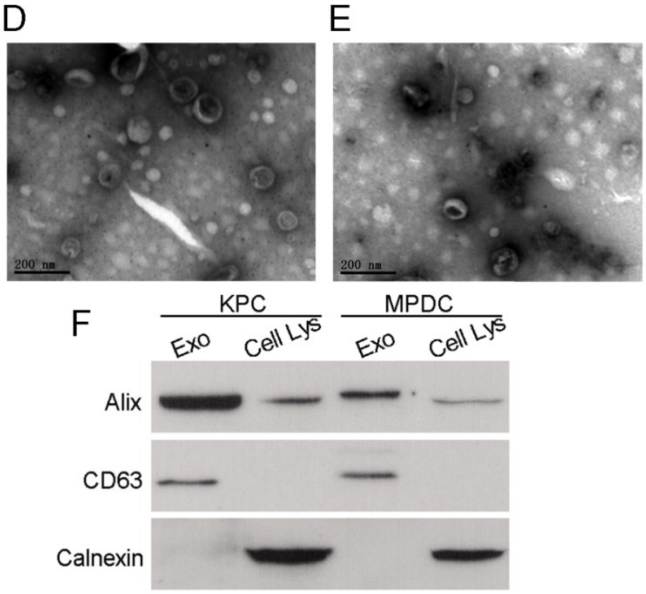

(A-C) Analysis of concentration and size distribution of exosomes by q-Nano particle analyzer. (D&E) Electron microscopy of exosomes. Scale bar, 200nm. (F) Western blot for exosomes protein marker (Alix, CD63). Calnexin used as a negative control.

Figure S2. The differentiation process of C2C12 myoblasts.

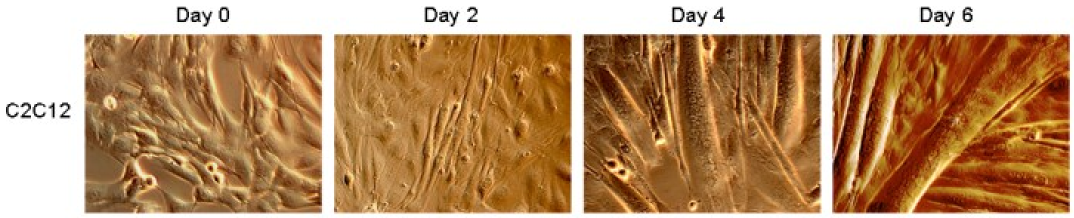

When the medium was replaced by differentiation medium, the morphological characterization of C2C12 myoblasts began to change. On the 1st Day, cells were fusiform or irregular triangle, with 80% cell fusion. On the 2nd day, cells were plurinuclear and still fibroblast-like. On the 4th Day, the formation of multinucleated myotubes formed. On the 6th day, mature C2C12 myotube cells were distinct. Images were captured at 40× magnification.

Figure S3. The effect of KPC-microvescicles on the insulin and PI3K/Akt signalling pathways.

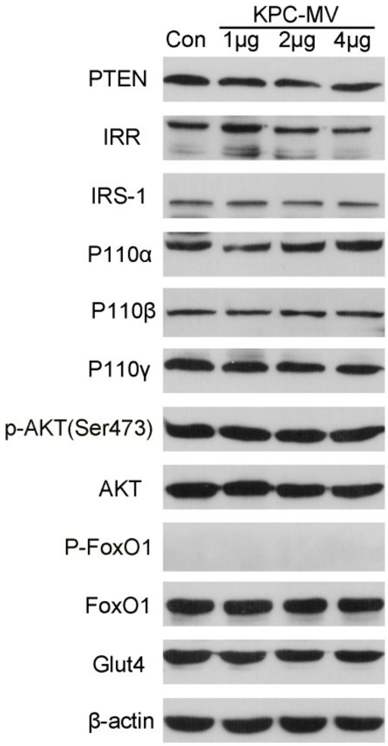

Differentiated C2C12 cells were cultured in 25-cm^2 culture-flasks with 5ml DMEM medium, and treated by KPC-microvescicles (1μg, 2μg, 4μg) for 24h.

Figure S4. Gene ontology analysis of the significant predicted genes of differently expressed miRNAs.

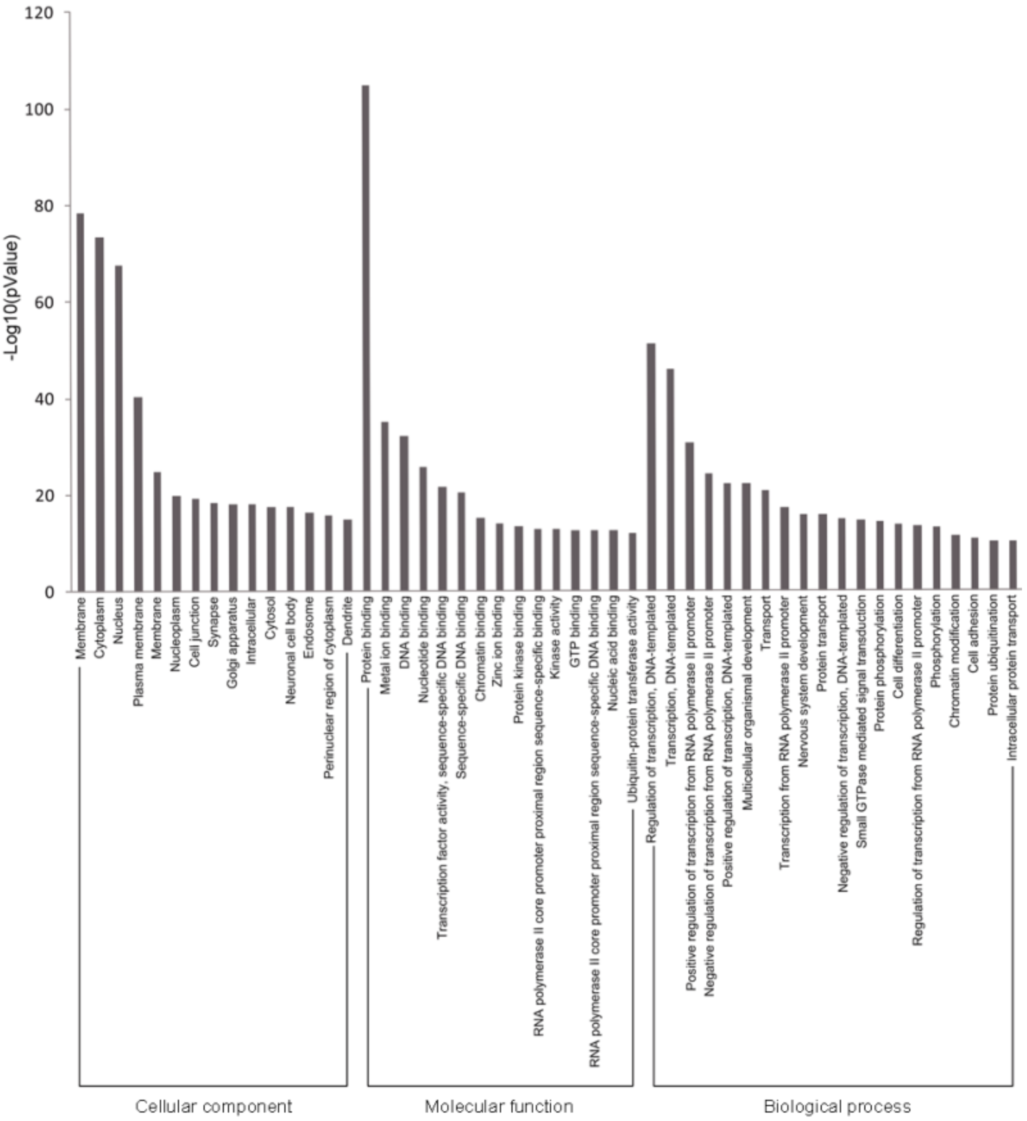

The results were classified into three main categories: cellular component, molecular function and biological process. The vertical axis indicated the significance of genes expression in each category. The horizontal axis showed the genes enrichment according to GO analysis.

Figure S5. Network among the PI3K/Akt, FoxO and insulin signalling pathways -related miRNAs and predicted genes.

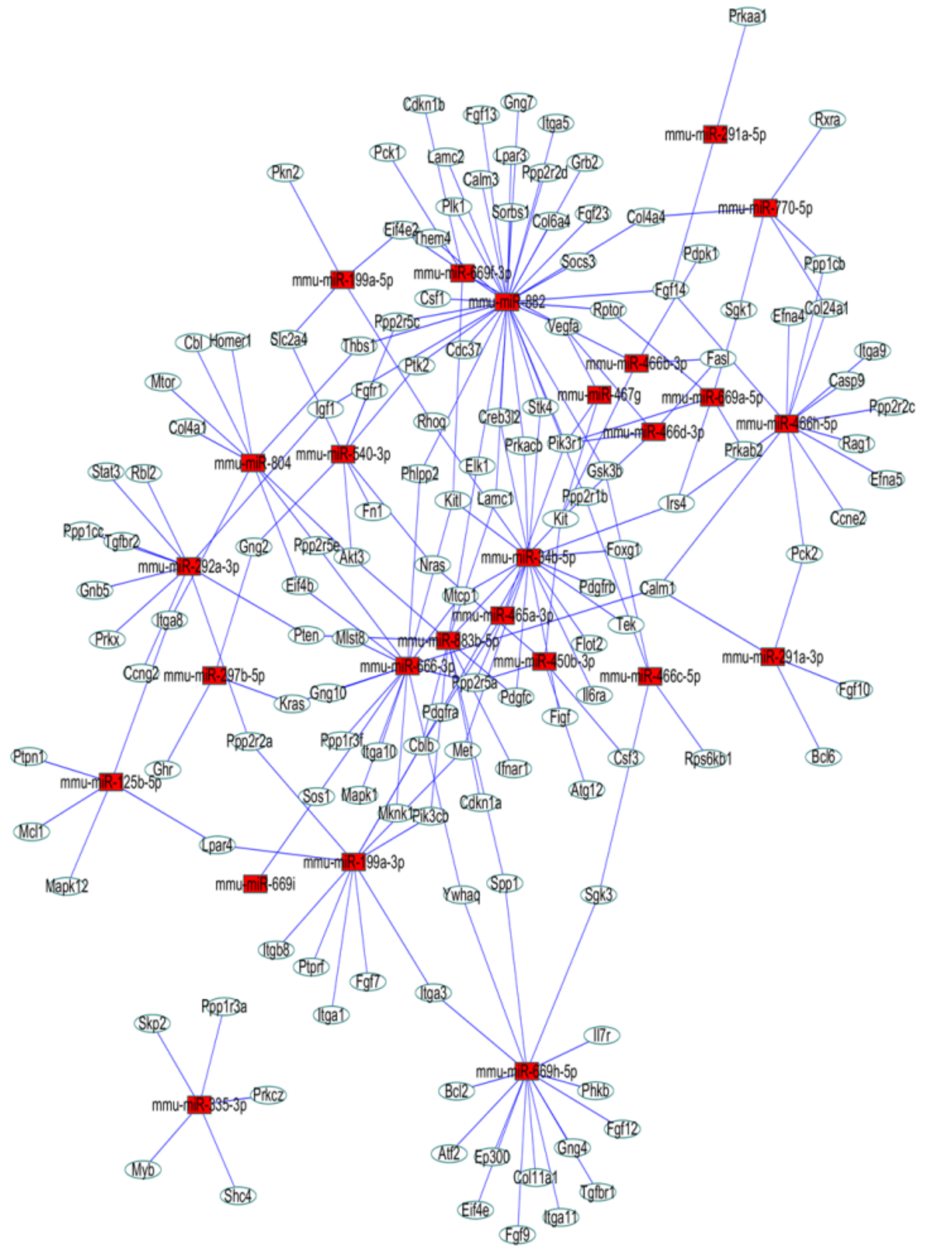

Squares represent miRNAs; Circles represent potential target genes; Straight lines represent possible relationship between miRNAs and predicted genes belonged to the 3 signalling pathways.

Figure S6. The autophagy induced by KPC-exosomes and insulin.

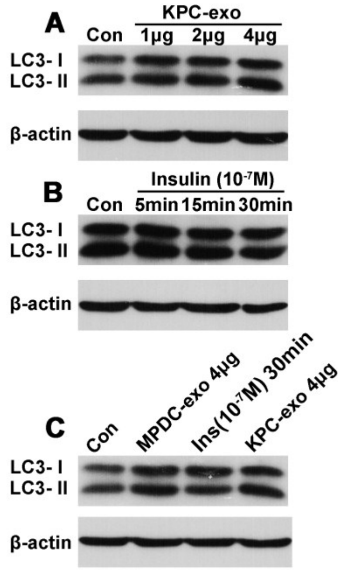

Though the expression of total amount of LC3 showed visible difference, the ratio of LC3II/I (LC3 II/LC3 I) showed no apparent difference in all the 3 groups (A,B,C).

Table 1. The primer sequences of miRNAs and U6.

	Primer	Sequences(5′-3′)
Highly expressed	mmu-miR-883b-5p	ACACTCCAGCTGGGTACTGAGAATGGGTAG
	mmu-miR-666-3p	ACACTCCAGCTGGGGGCTGCAGCGTGATCG
	mmu-miR-770-5p	ACACTCCAGCTGGGAGCACCACGTGTCTGG
	mmu-mir-804	ACACTCCAGCTGGGTGTGAGTTGTTCCTCA
	mmu-miR-540-3p	ACACTCCAGCTGGGAGGTCAGAGGTCGA
	mmu-miR-882	ACACTCCAGCTGGGAGGAGAGAGTTAGCGC
	mmu-miR-125b-5p	ACACTCCAGCTGGGTCCCTGAGACCCTAAC
	mmu-miR-450b-3p	ACACTCCAGCTGGGATTGGGAACATTTTGC
	mmu-miR-151-3p	ACACTCCAGCTGGGCTAGACTGAGGCTCC
Lowly expressed	mmu-miR-466b-3p	ACACTCCAGCTGGGATACATACACGCACAC
	mmu-miR-669h-5p	ACACTCCAGCTGGGATGCATGGGTGTATAGTT
	mmu-miR-199a-3p	ACACTCCAGCTGGGACAGTAGTCTGCACAT
	mmu-miR-466c-5p	ACACTCCAGCTGGGTGATGTGTGTGTGCATGT
	mmu-miR-466d-3p	ACACTCCAGCTGGGTATACATACACGCAC
	mmu-miR-292a-3p	ACACTCCAGCTGGGAAAGTGCCGCCAGGTTTT
	mmu-miR-291a-5p	ACACTCCAGCTGGGCATCAAAGTGGAGGCC
	mmu-miR-669a-5p	ACACTCCAGCTGGGAGTTGTGTGTGCATGTTC
	mmu-miR-34b-5p	ACACTCCAGCTGGGAGGCAGTGTAATTAGCT
endogenous control	U6-F	CTCGCTTCGGCAGCACA
	U6-R	AACGCTTCACGAATTTGCGT
